# Assessing Pain Levels Using Bioelectrical Impedance in Low Back Pain Patients: Clinical Performance Evaluation

**DOI:** 10.3390/diagnostics14212447

**Published:** 2024-10-31

**Authors:** Seungwan Jang, Jong Gab Ho, A-Ram Jo, Seung Mo Yoo, Hoonyoung Lee, Hyunyoung Lee, Young Kim, Se Dong Min

**Affiliations:** 1 Department of Software Convergence, Soonchunhyang University, Asan 31538, Republic of Korea; seungwanjang93@gmail.com (S.J.); hodori1988@sch.ac.kr (J.G.H.); athena1227@naver.com (H.L.); 2 Department of Business Administration, Kyung Hee University, Seoul 02453, Republic of Korea; blueriver724@naver.com (A.-R.J.); hylee@khu.ac.kr (H.L.); 3 Occupational and Environmental Medicine, Yesan Myongji Hospital, Yesan 32423, Republic of Korea; seoungmo@naver.com; 4 Department of Medical IT Engineering, Soonchunhyang University, Asan 31538, Republic of Korea

**Keywords:** bioelectrical impedance parameter, pain diagnosis, real time, treatment monitoring, musculoskeletal pain

## Abstract

Background/Objectives: Musculoskeletal pain is one of the leading causes of years lived with disability worldwide and has a negative impact on daily life and quality of life. Methods: The purpose of this study was to analyze the electrical characteristics of back pain by measuring and calculating bioelectrical impedance variables (R, Z, PA) in 85 subjects (45 in the Healthy group and 40 in the LBP group). Additionally, impedance measurements were conducted on 20 subjects (10 in the Young group and 10 in the Older group) to assess the impact of aging. Results: Bioelectrical impedance parameter values were higher in cases of back pain, and correlation analysis showed that there was a statistically significant difference between the Healthy and LBP groups (*p* < 0.05). A positive correlation was found between impedance parameters and pain related indices (ODI, RMDQ, VAS) (mean R, Z, PA: 0.68, 0.54, 0.75), with BMI positively correlating only with PA (0.493). Diagnostic accuracy for detecting back pain exceeded 95% (R, Z, PA: 0.984, 0.984, 0.963). Conclusions: Results indicated that aging did not significantly affect impedance values. The bioelectrical impedance measurement device used in this study, with its simultaneous diagnostic and therapeutic capabilities, proved useful for real-time pain diagnosis and treatment monitoring, highlighting its potential clinical utility.

## 1. Introduction

Musculoskeletal pain is one of the significant health issues negatively impacting daily life and quality of life [1]. Musculoskeletal pain can arise from various causes such as degenerative joint diseases due to aging, joint inflammation due to overuse, and idiopathic pain that is difficult to diagnose accurately. Among them, low back pain (LBP) is the most frequently occurring [2,3,4].

LBP is most frequently experienced between the L1 and L5 vertebrae levels of the lumbar spine [5], significantly impacting the quality of life due to limitations in lumbar curvature and difficulty in rotational movements caused by pain [2,3,6]. As a result, it is one of the major causes of years lived with disability (YLDs) worldwide [7,8].

YLDs is an indicator measuring the disability experienced over a specific period of life due to diseases or injuries negatively impacting health. Physical disabilities resulting from LBP have been identified as the most significant cause of YLDs compared to chronic conditions such as diabetes, chronic obstructive pulmonary disease, and other chronic illnesses [9].

To alleviate such pain, clinical approaches include pain management modalities such as electrical stimulation, muscle relaxation/inflammatory medication therapy, therapeutic exercise, and postural correction through rehabilitation physiotherapy. Among these, transcutaneous electrical nerve stimulation (TENS) is a non-invasive electrical therapy that stimulates peripheral nerves through skin surface electrodes, delivering therapeutic currents to the muscle fibers safely. This is a validated treatment technique effective for both acute and chronic pain management regardless of the pain’s etiology [10,11].

Diagnosis and treatment of pain are distinguished based on their intended purposes. Consequently, devices that simultaneously perform both diagnosis and treatment are uncommon. To address such limitations, the utilization of digital health technology for continuous measurement, diagnosis, and treatment of pain is gaining attention [12]. Objective and quantitative physiological data collected using digital devices are being utilized to develop digital biomarkers. However, various studies involving pain patients are needed to validate the clinical effectiveness of digital biomarkers [13].

Pain is known to be only assessed through the subjective descriptions provided by patients, and it is understood that there are no biomarkers available for accurately diagnosing it [14]. Particularly, nociceptive pain involves a dynamic interplay of various physiological mechanisms, where peripheral and central nervous systems, as well as psychological triggering, are all intertwined, making it extremely challenging to separate and evaluate them. Consequently, there continues to be a persistent demand for medical devices that can objectively and scientifically quantify and diagnose pain [15].

Recent research has been conducted to investigate or elucidate the electrical characteristics of pain using bioelectrical impedance to objectify pain [16,17,18]. According to research findings, it has been confirmed that subjective pain assessments can be quantified through the measurement and analysis of human skin bioelectrical impedance. The sympathetic electrical activity of the skin nerves is detected by surface electrodes. Additionally, variations in electrical signals are known to provide information about physiological and electrochemical phenomena related to mental stress, sweating, or pain [19]. In clinical settings, bioelectrical impedance is widely used for quantitative evaluation of pain due to the need for higher-quality pain management and monitoring [20].

Bioelectrical impedance is used to detect various pathological and physiological phenomena by monitoring physiological processes and tissue dynamics. It is employed in monitoring wound healing and swelling processes and evaluating the health of muscles affected by musculoskeletal and neuromuscular disorders [21,22,23]. Bioelectrical impedance involves injecting sinusoidal currents into muscle tissues to allow current flow along muscle fibers, thereby measuring the voltage drop in muscles caused by tissue impedance [24]. In the case of LBP, the function of the lumbar multifidus muscle is impaired due to a decrease in the muscle fibers of the lumbar multifidus muscle that maintains the stability of the lower back and fatty infiltration in which fatty tissue accumulates, which weakens the stability of the lower back and causes pain [25,26]. Fatty infiltration causes inflammation, which increases the sensitivity of nerve cells and makes them more sensitive to pain. The inflammatory response affects the ion channels of nerve cells, making them more responsive to pain and making the nerves more easily stimulated [27]. However, research findings on the electrical characteristics of pain, by pain location and cause, bioelectrical impedance depending on the presence of pain, and the accuracy of pain indexes/parameters, are still limited.

In this study, bioelectrical impedance was measured to assess the electrical characteristics based on the presence or absence of LBP; by analyzing this the features of bioelectrical impedance parameters (BIP) were compared according to the presence or absence of pain. Also, the accuracy of BIP in assessing LBP was confirmed, and the correlation between subjectively perceived pain degree and BIP was analyzed.

## 2. Methods

### 2.1. Subject Characteristics

In this study, we analyzed the BIP of the lumbar paraspinal muscle (LPM) according to age and the BIP difference and diagnostic accuracy according to the presence or absence of LBP in 105 participants. First, to confirm the difference in impedance by age, a total of 20 participants were recruited, including 10 into the Young group without LBP (28.2 ± 5.9 years old, 5 men, 5 women; male to female ratio of 1) and 10 into the Older group (54.9 ± 4.72 years old, 5 men, 5 women; male to female ratio of 1) (Table 1). Second, to perform a cross-sectional analysis of the diagnostic accuracy characteristics of LBP, a total of 85 participants were recruited, including 45 into the Healthy group (26.16 ± 5.90 years old, 19 men, 26 women; male to female ratio of 0.73) and 40 into the LBP group (59.15 ± 14.75 years old, 22 men, 18 women; male to female ratio of 1.22) (Table 2).

The sample size of the cross-sectional study subjects was determined using G-Power software 3.1.9.7. according to Cohen’s criteria, with a confidence level of 95% and a power of 0.80 for the receiver operating characteristic (ROC) curve analysis [28]. When the criterion for the ROC curve results was set to 0.80, it was calculated that 72 subjects would be needed to detect statistically significant effects. Considering a dropout rate of 10%, it was deemed appropriate to recruit a total of at least 80 subjects (including both the Healthy and LBP groups).

The recruitment criteria for the comparative evaluation of impedance by age included healthy adults aged 20 to 35 years (Young group) who did not feel discomfort during electrical stimulation, had no history of lumbar spine surgery, and did not experience LBP, as well as healthy adults aged 50 to 70 years (Older group).

For conducting a cross-sectional analysis of the diagnostic accuracy characteristics of LBP, a separate group of healthy adults aged 20 to 50 years (Healthy group) was recruited, who met the same criteria. The LBP group consisted of 40 outpatients diagnosed with LBP to evaluate the electrical characteristics of the lumbar region. Individuals in the LBP group with diseases such as polyneuropathy, mononeuropathy, and patients with a history of spinal surgery were excluded. Information about the subject of recruitment was disseminated through notices posted at the hospital and on campus.

The procedure for obtaining the subjects’ consent to participate in the study involved verbal explanation followed by the completion of written informed consent forms by the research subjects. This study received approval from the Institutional Review Board (IRB Approval Number: 1040875-202303-SB-016).

### 2.2. Low Back Pain Evaluation Form and Body Mass Index

LBP severity was evaluated using the visual analogue scale (VAS), which visualizes information on functional disability and pain. VAS is a scale that represents the degree of pain visually, with 0 indicating ‘no pain’ and 10 indicating ‘unbearable pain’. The Korean version of the Oswestry Disability Index (ODI) was used as an evaluation sheet to evaluate spinal disease [29]. The ODI consists of 10 items assessing the level of pain and disability in various physical activities. Each question is rated on a six-point scale (0–5), where 0 represents “no limitation”, and 5 represents “severe limitation”. The second assessment tool used was the Korean version of the Roland–Morris Disability Questionnaire (RMDQ) [30]. The RMDQ is a method for evaluating disability caused by LBP in clinical settings, consisting of 24 questions. Scores are calculated based on the number of selected questions, with higher scores indicating more severe pain.

BIP measures the current flow through and returning from the skin and various layers of tissue (fat, muscle, etc.) beneath the skin. In this process, the electrical resistance characteristics of the skin and internal tissues are measured. In the case of LBP, fat tends to be more than muscle. Fat has the characteristic of impeding the flow of current more than muscle, so the measured BIP value increases. To confirm this, body mass index (BMI), which represents body composition, was calculated using the subject’s height and weight (kg/m^2^). This study was conducted at Yesan Myongji Hospital from June 2023 to June 2024.

### 2.3. Bioelectrical Impedance Measuring Equipment

In this study, Pain Bot (Red & Blue Co., Ltd., Yesan, Republic of Korea) was used to measure the electrical characteristics of the LPM. This device uses a bipolar configuration with a probe for supplying alternating current (AC) and an electrode for measurement [23,31] and can perform low-frequency treatment and pain site diagnosis simultaneously. Pain Bot operates within the frequency range of 182 Hz, with the output current set at 50 mA [32,33]. For low-frequency therapy and diagnosis, the voltage can be adjusted from 1.5 V to 30 V in 0.5 V increments, and pulse intervals can be set at 250 μs, 500 μs, or continuous modes. However, in this study, to minimize therapeutic effects and observe changes in measurement values for diagnostic purposes, the voltage was set at 3.5 V with continuous pulse mode during the measurement process.

**Figure 1:**
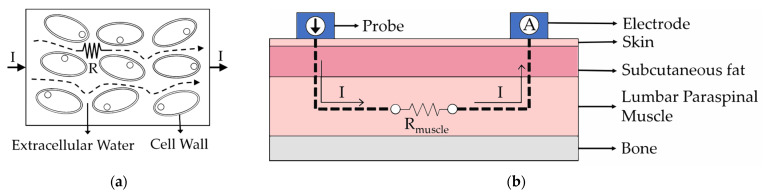
Bioelectrical impedance measurement principle: (**a**) low frequency resistance measurement; (**b**) LPM measurement with probe and electrode.

Although the 182 Hz frequency does not penetrate the cell membrane, it flows along the cell wall (Figure 1a) and provides important information about the structure and composition of tissues and the temporal relaxation processes of cells [31]. Electrical impedance theory is a theory that describes the resistance and response of tissues when current passes through them. BIP measurements based on this theory typically measure tissue resistance using low currents (μA) and high frequencies (>1 kHz). In contrast, the TENS technology-based device used in this study applies high currents (mA) and low frequencies (<200 Hz). Although 182 Hz is considered a low frequency for BIP measurements, the high current allows the signal to pass through the subcutaneous fat layer and reach the muscle tissue [34].

Therefore, the resistance value at the measurement site varies depending on the pain condition, such as myofascial pain syndrome, muscle tension, inflammation, or fat infiltration [23,31,35].

Due to the change in resistance value depending on the pain condition, the voltage set at the probe (3.5 V) and the voltage measured at the disposable pad vary according to the resistance values of the tissues altered by pain (Figure 1). This indicates that the changing resistance values can be measured using Ohm’s law, V = IR.

### 2.4. Experimental Protocol

This study was conducted in a spine clinic in a hospital. Data collection of BIP for the LBP group and the Healthy group was performed by measuring the LPM area between L1 and L5. All subject data collection was conducted by one occupational medicine specialist with over 30 years of experience. Prior to participating in the experiment, all subjects were informed about the research overview and ethical considerations based on documents approved by the IRB. Subjects volunteered to participate and joined the study after fully understanding the purpose of the experiment, its procedures, potential risks, and benefits of participation.

**Figure 2:**
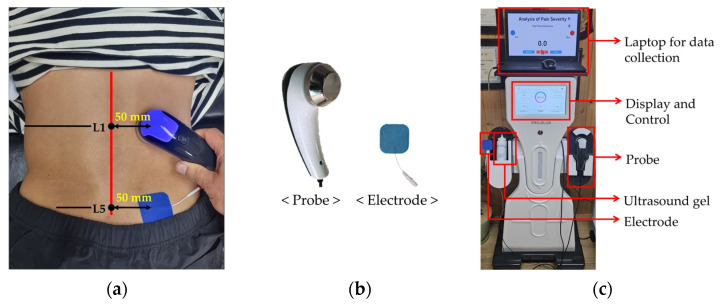
Bioelectrical impedance measurement method and equipment: (**a**) probe and electrode attachment sites; (**b**) probe and electrode; (**c**) bioelectrical impedance measurement device.

The subjects first completed the ODI, RMDQ, and VAS questionnaires, and then measured waist circumference at the midline of L1–L5 using a ruler. The subjects were then placed in the prone position on the physiotherapy bed, and the skin area where the probe and electrodes were to be attached was sterilized with an alcohol swab and coated with ultrasound gel. The electrode was attached 50 mm horizontally from the L5 spinous process, and the probe was in contact with the L1 spinous process in the same direction as the attached electrode, also located 50 mm horizontally (Figure 2a).

Measurements were repeated three times for 15 s each, with a 30 s rest period between each measurement, to ensure data accuracy and consistency. After completing three measurements on one side, the electrode and probe were attached to the other side using the same method, and they were contacted three times for 15 s each, for a total of 90 s, repeated six times.

### 2.5. Bioelectrical Impedance Parameter Calculation

**Figure 3:**
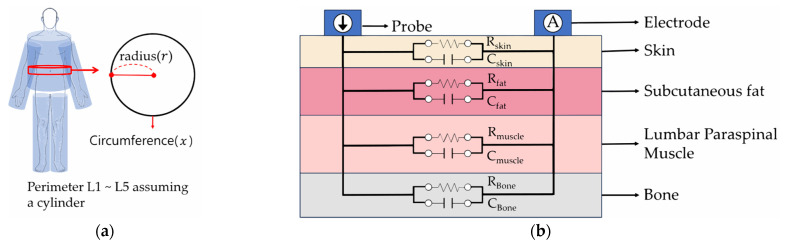
Human body model for calculating capacitance in bioelectrical impedance: (**a**) Cylindrical human body model used for bioelectrical impedance analysis; (**b**) circuit model of electrical/skin contact, epidermal and subcutaneous impedance.

The bioelectrical impedance measurement device used in this study employs two electrodes to measure the resistance value of the pain site [23,31]. This technology follows Ohm’s law, allowing the extraction of variables such as resistance (R), impedance (Z), and phase angle (PA) based on the amplitude of the injected current and recorded voltage signals [23,31]. The capacitance values of the human body calculated in this study were used to derive Z and PA, which are parameters indicating the severity of pain. Capacitance is the ability to store electricity, which is the ability of a material to store and maintain an electric charge. In biological tissues, especially in the human body, capacitance occurs due to the cell structure and the presence of ionic fluids. Measurement method of this study, the capacitance of the human body can be assumed to be connected in parallel as in Figure 3b. In this case, the total capacitance can be calculated as the sum of the capacitances of each biological tissue.

When R increases in the LPM area, the flow of current is impeded, reducing the ability to store electrical energy and resulting in lower capacitance values. Z refers to the sum of R and reactance that occurs when an electrical signal flows, and as the R of the LPM area increases, the Z value also increases. PA is related to the speed of electrical signal transmission and represents the phase difference between voltage and current in AC circuits. In cases of inflammation or increased fat in the LPM area, electrical signal transmission slows down, leading to a decrease in PA. To calculate the capacitance value, we used the values of the permittivity of tissues (ε) (skin: 1.14 × 10^3^, fat: 7.20 × 10^4^, muscle: 4.22 × 10^6^, blood: 5.26 × 10^3^, bone: 7.82 × 10^4^ and connective tissue: 6.11 × 10^6^) at the Pain Bot low-frequency output frequency of 182 Hz [36] and used the ratio (ω) (Skin: male and female 15%, fat: male 20%, female 30%, muscle: male 42%, female 38%, blood: male and female 8%, bone: male and female 7%, connective tissue: male 8%, female 2%) of body tissues as in Equation (1) [37,38] to calculate the permittivity of each tissue. These values were then summed to determine the overall permittivity ($\varepsilon_{r}$). When calculating $\varepsilon_{r}$, we used different ratios for males and females. Based on the cylindrical model of the human body used in bioelectrical impedance analysis, we assumed the waist circumference (x) between L1 and L5 of each subject was assumed to be the circumference of a circle, calculated the radius (r) using Equation (2), and then calculated the Area (Figure 3a). For the distance calculation, we utilized the gap value between L1 and L5, and the vacuum permittivity of free space as $\varepsilon_{0}[F/m]=8.855\times 10^{-12}$ (Equation (3)).

(1) $$\varepsilon_{r}=\frac{\omega_{skin}}{\varepsilon_{skin}}+\frac{\omega_{fat}}{\varepsilon_{fat}}+\frac{\omega_{muscle}}{\varepsilon_{muscle}}+\frac{\omega_{bone}}{\varepsilon_{bone}}+\frac{\omega_{blood}}{\varepsilon_{blood}}+\frac{\omega_{connective\;Tissue}}{\varepsilon_{connective\;Tissue}}$$

(2) $$r=\frac{x}{2\pi },\;\mathrm{Area}=\pi \times r^{2}$$

(3) $$C=\varepsilon_{0}\varepsilon_{r}\frac{\mathrm{Area}}{\mathrm{Distance}}\;[F]$$

Using the calculated capacitor values, Z was computed (Equation (4)), where R represents the R value measured from the subjects LPM area, and f denotes the low-frequency output frequency of the measuring equipment, 182 Hz. PA was calculated using Equation (5) [39]. Subsequently, to minimize data errors, the average of left and right data was utilized.

(4) $$Z=\sqrt{\frac{1}{\frac{1}{R^{2}}+{\left(2\pi \mathrm{fC}\right)}^{2}}}\;$$

(5) PA=−arctan(2πfCR)

## 3. Results

### 3.1. Comparative Analysis of BIP by Age Group Using T-Test

**Figure 4:**
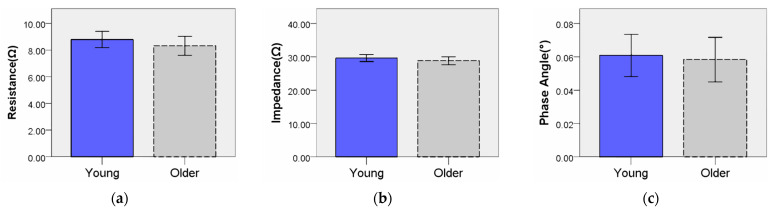
Differences in BIP by age group: (**a**) resistance; (**b**) impedance; (**c**) phase angle.

An independent sample *t*-test was used to compare the differences in BIP by age group. This method is effective for comparing mean differences between two groups and evaluating statistical significance. The subjects were divided into 20–35 years old (Young group) and 50–70 years old (Older group). Table 3 summarizes the results of the independent sample *t*-test performed to confirm the differences in BIP values by age group. Figure 4 depicts a graph with error bars set at 95%. The analysis results showed R (t = 1.121, *p* > 0.05), Z (t = 1.148, *p* > 0.05), PA (t = 0.301, *p* > 0.767), and the *p* values of all BIP variables were greater than 0.05, so the null hypothesis was adopted. In other words, the differences in BIP parameters by age group were not statistically significant, showing that there was no difference in BIP values by age group.

### 3.2. Comparative Analysis of T-Test According to the Presence or Absence of Low Back Pain

**Figure 5:**
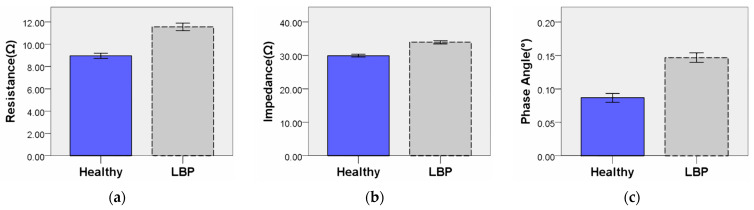
Differences in BIP between Healthy and LBP groups based on the presence or absence of LBP: (**a**) resistance; (**b**) impedance; (**c**) phase angle.

To assess the difference in BIP on the presence of LBP, an independent sample *t*-test was used. The analysis results, presented in Table 4, summarize the results of the independent sample *t*-test conducted to examine the correlation between BIP according to the presence of LBP. Figure 5 depicts a graph with error bars set at 95%. The analysis revealed that BIP values, including R (t = 12.45, *p* < 0.05), Z (t = 12.55, *p* < 0.05) and PA (t = 12.63, *p* < 0.05), showed statistically significant results based on the presence of LBP.

### 3.3. Accuracy Analysis of Quantitative Measurements by BIP

**Figure 6:**
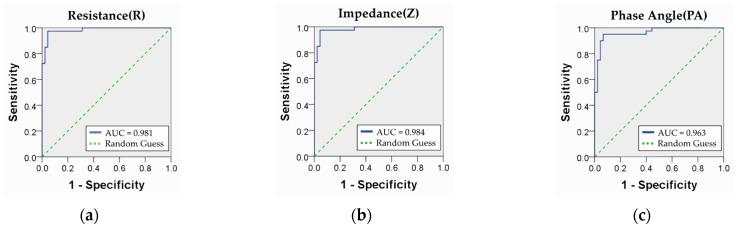
ROC curve for pain diagnostic ability: (**a**) resistance; (**b**) impedance; (**c**) phase angle.

The accuracy of determining the presence of LBP using the measured BIP (R, Z, PA) was evaluated using the ROC curve. The ROC curve illustrates the relationship between the False Positive Rate (FPR) and True Positive Rate (TPR). It is commonly used to assess the utility of diagnostic tools and evaluate the accuracy of tests, as well as to establish the threshold (cut point) for tests in diagnostic tool development. According to the analysis results, the prediction accuracies for the presence or absence of LBP were 0.981, 0.984, and 0.963, respectively. The sensitivity was 0.950 for R and Z, and 0.925 for PA, and the specificity was 0.956 for R and Z and 0.933 for PA (Figure 6).

The optimal cutoff points for distinguishing the presence of LBP based on the scores of each variable were determined through ROC analysis, as presented in Table 5. The Youden Index was utilized as the cut-off point estimation metric, and cut-off points of 10.09 Ω, 31.89 Ω, and 0.12° were adopted for R, Z, and PA, respectively.

### 3.4. Correlation Analysis Between BIP and ODI, RMDQ, VAS, BMI

To analyze the correlation between the severity and degree of LBP, as indicated by ODI, RMDQ, VAS, and BMI representing body fat index, as well as the electrical characteristic represented by BIP, Pearson correlation analysis was conducted. Pearson correlation analysis is a statistical method used to evaluate the linear relationship between two variables. In this study, it was employed to assess the correlation between pain indicators and BIP. The results of the analysis are summarized in Table 6, demonstrating positive correlations between R and ODI, RMDQ, VAS (r = 0.668, r = 0.511, r = 0.761, *p* < 0.01), as well as between Z and ODI, RMDQ, VAS (r = 0.688, r = 0.516, r = 0.762, *p* < 0.01). Additionally, PA showed positive correlations with ODI, RMDQ, VAS, and BMI (r = 0.677, r = 0.612, r = 0.748, r = 0.493, *p* < 0.01).

### 3.5. Quantification and Correlation Analysis of VAS and Low Back Pain

Based on the results of Pearson correlation analysis, the VAS score that showed the highest positive correlation with BIP among ODI, RMDQ, VAS, and BMI was mapped, and the correlation was analyzed. The range of collected VAS scores was 0 to 2 and 4 to 8. Among these, the Healthy group all had scores ranging from 0 to 2 and were treated as one group, and one patient with a VAS score of 2 was excluded. In addition, in the case of the LBP group, the VAS scores were classified into three groups, as shown in Table 7, to minimize errors due to the sample size of 10 or less in the VAS score category and to group close VAS scores into the same group [40].

To determine if there were statistically significant differences in BIP based on pain severity after mapping with VAS, one--way ANOVA was conducted and summarized in Table 8, Table 9 and Table 10. The analysis revealed statistically significant differences between group 0 and the other groups for all BIP, while no significant differences were observed among the other groups.

## 4. Discussion

In this study, we analyzed the impedance of LPM according to age, the impedance difference, and diagnostic accuracy according to the presence or absence of LBP in 105 participants. To confirm the effect of aging on BIP values, the bioelectrical impedance of 20 subjects (10 Young group, 20–35 years old, and 10 Older group, 50–70 years old) was measured and evaluated. In addition, the electrical characteristics (Z, PA) of LBP were calculated through the bioelectrical impedance (R) measurement of 85 subjects (45 in the Healthy group and 40 in the LBP group). The measured and calculated BIP were analyzed to categorize the presence of LBP. The correlation between the subjective perception of pain intensity (VAS), disability scores (ODI, RMDQ), BMI, and BIP was analyzed. Furthermore, the measurement accuracy of pain based on each BIP (R, Z, PA) was analyzed. The results indicated that aging did not have a statistically significant impact on the measured BIP values. Finally, we mapped the relationship between BIP and VAS, which had the highest positive correlation with BIP, and statistically confirmed it.

A study analyzing BIP, including R, Z, and PA based on the composition of the intracellular matrix in cells, has been conducted to analyze the characteristics of pain. LBP tends to exhibit higher values of R and Z compared to those in the Healthy group, while PA tends to be higher in the Healthy group [18].

However, it is important to consider that the 182 Hz frequency band used in this study has a greater contribution from the extracellular matrix (ECM) in bioimpedance measurements because the current does not easily pass through the cell membrane. The bioimpedance values measured at the 182 Hz frequency mainly reflect the characteristics of the extracellular space and ECM, which greatly affect the distribution of electric fields and ion movement [23,31]. Therefore, the increase in impedance observed in LBP patients is caused not only by changes in the intracellular matrix but also by structural changes in the ECM and changes in the distribution of body fluids. structural changes in the ECM (e.g., increased collagen or fibrosis) have a significant impact on low-frequency bioimpedance measurements by impeding the flow of current within the tissue. Collagen accumulation increases the density of the ECM, restricting the movement of ions, and fibrosis increases R by impeding the flow of current along with a decrease in tissue elasticity [23,31].

In addition, changes in the extracellular space, such as increased collagen accumulation or alterations in fluid distribution, contribute to differences in bioimpedance parameters. For example, fat infiltration, fibrosis, or inflammation of the ECM can increase tissue R and impedance, affecting the results of bioimpedance measurements [23,31]. Therefore, the differences in bioimpedance parameters between LBP and Healthy can be explained not only by changes in the intracellular matrix composition but also by alterations in ECM characteristics.

There are some important limitations to using a single frequency. This is because the characteristics of BIP at different frequencies vary depending on the cell membrane, ECM, and electrical environment. Considering the difference in impedance characteristics at low and high frequencies, low frequencies are greatly affected by the cell membrane and ECM, whereas high frequencies easily pass through the cell membrane and are relatively less affected by the ECM [23,31]. Therefore, when using a single frequency, it may be difficult to fully reflect the electrical characteristics of the tissue with only the impedance value that appears at a specific frequency. This may make it difficult to identify the actual cause of LBP, but LBP caused by changes in the ECM, such as fatty infiltration or inflammation, is sufficiently reliable. Therefore, if measurements using various frequencies are performed in parallel, it is thought that a wider range of impedance characteristics can be identified and information on the cause of LBP can be provided. In future studies, it will be important to perform a more comprehensive impedance analysis through an experimental design that includes multiple frequencies.

Compared with other studies, the results of this study revealed that R, Z, and PA values were all higher in the LBP group compared to the Healthy group (*p* < 0.05) [17,18]. Regarding PA, it is presumed that a limitation arises during the process of calculating it using the capacitance of the capacitor based on the circumference and distance. While the average circumference in the Healthy group was measured as 12.26 ± 2.4 cm, it was 14.18 ± 1.24 cm in the LBP group. When this was substituted into Equation (2), the value of area increased, indicating that the PA of the LBP group increased. Nevertheless, the results of this study confirmed that there was a statistically significant difference in PA for classifying the LBP group and the Healthy group.

In addition, the 182 Hz frequency does not penetrate cell membranes but provides information on the structure and composition of tissues and the time-dependent relaxation process of cells related to the severity of LBP. This shows that BIP measured at 182 Hz can effectively distinguish between Healthy and LBP groups. In addition, when used in conjunction with TENS, it will be possible to monitor the treatment effect in real-time by tracking bioelectrical changes during treatment.

Table 11 summarizes the ROC curve results of studies measuring BIP in the LPM area using different measurement devices. The results obtained using the measurement device in this study showed higher accuracy compared to other studies. This difference is presumed to be due to the difference in output frequency of each measurement device. As the output frequency decreases, it is inferred that the difference in BIP values between LBP and Healthy groups becomes more pronounced [17]. At low frequencies, the electrical properties of the cell membrane and the electrical properties of the extracellular space have a greater impact on the overall impedance. Low-frequency currents have difficulty passing through the cell membrane, so the electrical capacitance of the cell membrane and the influence of the ECM has a greater effect on impedance measurements [41]. On the other hand, at high frequencies, currents can easily pass through the cell membrane, so the influence of the ECM is less, and the impedance tends to be relatively low [31]. Therefore, if changes occur in the extracellular space due to inflammation, fatty infiltration, etc., the difference in BIP values becomes more evident.

The Pain Bot used in this study operates at a frequency range of 182 Hz, which explains its higher accuracy compared to that of other studies. Additionally, the cutoff points were determined using the Youden Index for R, Z, and PA (10.09 Ω, 31.89 Ω, 0.12°, respectively). An R-value of 10.09 Ω or higher indicates a high likelihood of diagnosing a LBP. Similarly, a Z-value of 31.89 Ω or higher indicates a high likelihood of diagnosing an LBP, and a PA-value of 0.12° or higher also indicates a high likelihood of diagnosing an LBP.

Table 12 compares the BIP values, including between the Healthy group and the LBP group in the present study and the reference study. The Healthy group showed lower values for R (8.97 Ω), Z (29.92 Ω), and PA (0.09), while the LBP group showed higher values for R (11.55 Ω), Z (33.97 Ω), and PA (0.15). These results suggest that there is a difference in BIP values between the two groups, and that low-frequency EBI measurements can effectively distinguish Healthy from LBP. In contrast, the reference studies by Wang et al. [17] and Wang et al. [18] used higher frequencies, where the contribution of intracellular components such as water and cell membranes is more prominent. Wang et al. [17] reported higher impedance values in both the Healthy (3.48 KΩ) and LBP (6.09 KΩ) groups. This difference is interpreted as being due to the different frequency ranges used in each study.

To evaluate the presence or absence of pain in the LBP group, pain questionnaires for VAS, ODI, and RMDQ were completed, and BMI was calculated using weight and height. Afterward, Pearson correlation analysis was performed to analyze the correlation with BIP. The analysis results (** *p* < 0.01) showed a positive correlation between all BIP and ODI (R: 0.688 **, Z: 0.688 **, PA: 0.677 **), RMDQ (R: 0.511 **, Z: 0.516 **, PA: 0.612 **), VAS (R: 0.761 **, Z: 0.762 **, PA: 0.748 **) and BMI (R: 0.192, Z: 0.195, PA: 0.493 **). Among them, VAS exhibited the highest positive correlation. This confirms a significant positive correlation between the level of LBP perceived by and the BIP measured at LPM. However, except for PA, there was no correlation between BMI and PA. This is believed to be a phenomenon that occurred using calculated BMI values. It was confirmed that the calculated BMI does not consider fat and muscle mass, and even if the actual BMI is high, pain does not always occur because muscle mass is sometimes high [42].

Through Pearson correlation analysis, it was confirmed that there is a strong positive correlation between VAS scores and BIP values. To statistically verify this, one-way ANOVA was conducted, showing a significant difference between VAS group 0 and groups 1, 2, and 3. However, no statistically significant differences were found in the remaining groups. This indicates a lack of statistical significance between the subjectively measured VAS scores and objectively measured BIP. In other words, it was revealed that an increase in VAS scores is not related to an increase in BIP values. This is inferred to be due to an error in the subjective measure VAS, where a score that should have been marked as 6 was recorded as 5, resulting in no increase in BIP values despite an increase in VAS scores [43]. Also, the lack of differentiation between acute and chronic pain has been noted. Chronic pain is defined as pain persisting for more than three months, which can lead to various changes in psychological and sensory aspects, causing a discrepancy between the actual severity of pain and the perceived intensity of pain. As a result, VAS scores may also decrease or increase [44]. To date, it has been confirmed that very few studies have attempted to quantify VAS scores and BIP values by mapping them. Most studies on objective measurement of pain based on VAS scores were conducted through comparisons before and after treatment or rehabilitation [39,45]. Previous studies have shown that objective indicators decrease as VAS scores decrease. The results of this study also showed that objective indicators tend to increase and decrease as subjective indicators increase and decrease. This suggests that there is a correlation between subjective and objective indicators. However, subjective indicators measured psychologically and sensorily and objective indicators measured physiologically may not exactly match due to various factors, and this discrepancy may result from the complex multidimensional nature of pain, differences in individual pain perception, or the influence of subjective experience on physiological responses [46]. For example, even when experiencing the same pain intensity, VAS scores may vary depending on an individual’s psychological state or emotional response, which may weaken the correlation with objective indicators. Therefore, understanding the differences between subjective and objective indicators will be a crucial factor in increasing the accuracy and validity of pain assessment. The distinction between the normal group and the LBP group can be based on the BIP, but it is difficult to distinguish the pain differences between the LBP groups. To solve this, research is needed that collects and analyze data by measuring pain levels over the long term. This approach will strengthen the relationship between subjective and objective indicators and enable more reliable evaluation in pain prognosis management and diagnosis.

In this study, acute and chronic pain were not separately considered but classified as one LBP group for analysis. Due to the characteristics of the hospital and region, there were limitations in recruiting patients, as many were elderly or had occupations involving prolonged standing or frequent use of the back, making it difficult to distinguish between chronic and acute conditions. This limitation arises from not considering the diversity of pain causes, pathology, and physiology, which could reduce diagnostic accuracy. However, in this study, the diagnostic accuracy was found to be over 95%, indicating that these limitations did not significantly affect accuracy but rather influenced the pain score. In future work, we plan to classify acute and chronic patients and recruit patients according to VAS scores to perform a clear and accurate analysis of more pain types and pain levels.

## 5. Conclusions

In this study, we calculated and identified the electrical characteristics (Z, PA) of LBP through bioelectrical impedance (R) measurements in the LPM of 85 subjects. In addition, the impedance change according to age was analyzed for 20 subjects to evaluate the effect of aging on bioelectrical impedance. As a result, it was found that aging did not significantly affect the results of bioelectrical impedance measurement. The BIP of 85 subjects was analyzed to classify the presence or absence of pain, and the accuracy of BIP measurement was evaluated through the ROC curve.

As a result of analyzing the correlation between subjective pain intensity (VAS), disability level (ODI, RMDQ), BMI, and BIP, a strong positive correlation was confirmed between the VAS score and BIP. However, the BIP value did not increase consistently as the VAS score increased, but it was confirmed to be useful in distinguishing the Healthy group from the LBP group. The results of this study suggest that BIP measurement can be an effective index for pain assessment. The low-frequency bioelectrical impedance device used in this study can provide information on the degree of LBP by measuring information on the structure and composition of tissues and the relaxation process of cells over time, despite not being able to penetrate cell membranes by using 182 Hz. Therefore, it can be used in conjunction with TENS to confirm treatment in real-time, and it has the advantage of monitoring bioelectrical changes occurring during treatment and evaluating the immediate effect of treatment.

In conclusion, this study measured the resistance value of LPM according to LBP based on the electrical impedance theory. By applying the frequency and current used in TENS, it was shown that the accuracy of bioelectrical impedance measurement can be effectively proven in evaluating low back pain. The results of this study suggest the possibility of quantitatively assessing pain in real-time and laying the foundation for future integrated treatment and monitoring studies. However, this study has limitations in distinguishing acute and chronic pain and classifying them into clear LBP groups, and it is difficult to identify the cause of LBP because a single frequency was used. In future studies, multiple frequencies should be used to analyze the electrical properties of biological tissues in more detail and identify the characteristics of various pain types. In addition, long-term studies are needed to confirm the effectiveness of real-time treatment and diagnostic monitoring.

## Figures and Tables

**Table 1:** Subjects information for analysis of impedance differences by age.

	Age ± SD	Height ± SD (cm)	Weight ± SD (kg)	BMI ± SD (kg/m^2^)	N
Young	28.2 ± 5.9	167.68 ± 9.4	66 ± 16.44	23.15 ± 3.62	10
Older	54.9 ± 4.72	164.4 ± 9.41	62.7 ± 15.07	22.88 ± 3.21	10

**Table 2:** Subjects information for cross-sectional study.

	Age ± SD	Height ± SD (cm)	Weight ± SD (kg)	BMI ± SD (kg/m^2^)	N
Healthy	27.16 ± 5.9	167.64 ± 9.03	63.13 ± 13.03	22.32 ± 3.41	45
LBP	60.1 ± 14.94	163.63 ± 9.28	66.4 ± 11.39	24.61 ± 2.92	40

**Table 3:** Comparison of differences in BIP by age group.

		N	Average	SD	t	*p*
BIP	Young R (Ω)	10	8.79	±0.87	1.121	0.277
	Older R (Ω)	10	8.33	±0.99		
	Young Z (Ω)	10	29.62	±1.46	1.148	0.266
	Older Z (Ω)	10	28.81	±1.69		
	Young PA (°)	10	0.061	±0.018	0.301	0.767
	Older PA (°)	10	0.058	±0.019		

R: resistance, Z: impedance, PA: phase angle.

**Table 4:** Comparison of differences in BIP according to the presence or absence of LBP.

		N	Average	SD	t	*p*
BIP	Healthy R (Ω)	45	8.97	±0.81	12.45 *	0.00
	LBP R (Ω)	40	11.55	±1.09		
	Healthy Z (Ω)	45	29.92	±1.37	12.55 *	0.00
	LBP Z (Ω)	40	33.97	±1.58		
	Healthy PA (°)	45	0.09	±0.022	12.63 *	0.00
	LBP PA (°)	40	0.15	±0.023		

* *p* < 0.05, R: resistance, Z: impedance, PA: phase angle.

**Table 5:** Area under the ROC curve for the ability to predict pain for bioimpedance with and without LBP.

	Cut-Off Value	Youden’s J	Sensitivity	Specificity	Accuracy
R	10.09 (Ω)	0.906	0.95	0.956	0.984
Z	31.89 (Ω)	0.906	0.95	0.956	0.984
PA	0.12 (°)	0.858	0.925	0.933	0.963

**Table 6:** Pearson correlation between BIP and evaluation papers.

BIP	ODI	RMDQ	VAS	BMI
R	0.688 **	0.511 **	0.761 **	0.192
Z	0.688 **	0.516 **	0.762 **	0.195
PA	0.677 **	0.612 **	0.748 **	0.493 **

** *p* < 0.01.

**Table 7:** VAS score group.

	Group 0	Group 1	Group 2	Group 3
VAS score	0–2	4–5	6–7	8
N	45	12	15	12

**Table 8:** Analysis of resistance value differences by VAS group.

VAS Group (I)	VAS Group (J)	Mean Difference (I-J)	SD	*p*-Value	95% Confidence Interval	
					Lower Bound	Upper Bound
R Group 0	R Group 1	−2.57 *	0.31	0.000	−3.44	−1.69
	R Group 2	−2.37 *	0.28	0.000	−3.17	−1.56
	R Group 3	−2.96 *	0.31	0.000	−3.83	−2.08
R Group 1	R Group 0	2.57 *	0.31	0.000	1.69	3.44
	R Group 2	0.2	0.37	0.958	−0.84	1.25
	R Group 3	−0.39	0.39	0.798	−1.49	0.71
R Group 2	R Group 0	2.37 *	0.28	0.000	1.56	3.17
	R Group 1	−0.2	0.37	0.958	−1.25	0.84
	R Group 3	−0.59	0.37	0.460	−1.63	0.45
R Group 3	R Group 0	2.96 *	0.31	0.000	2.08	3.83
	R Group 1	0.39	0.39	0.798	−0.71	1.49
	R Group 2	0.59	0.37	0.460	−0.45	1.63

* *p* < 0.05, R: resistance, group 0 = VAS 0–2, group 1 = VAS 4–5, group 2 = VAS 6–7, group 3 = VAS 8.

**Table 9:** Analysis of impedance value differences by VAS group.

VAS Group (I)	VAS Group (J)	Mean Difference (I-J)	SD	*p*-Value	95% Confidence Interval	
					Lower Bound	Upper Bound
Z Group 0	Z Group 1	−4.02 *	0.48	0.000	−5.40	−2.65
	Z Group 2	−3.71 *	0.44	0.000	−4.97	−2.46
	Z Group 3	−4.58 *	0.48	0.000	−5.95	−3.21
Z Group 1	Z Group 0	4.02 *	0.48	0.000	2.65	5.39
	Z Group 2	0.31	0.57	0.962	−1.33	1.94
	Z Group 3	−0.56	0.60	0.838	−2.28	1.17
Z Group 2	Z Group 0	3.71 *	0.44	0.000	2.46	4.97
	Z Group 1	−0.31	0.57	0.962	−1.94	1.33
	Z Group 3	−0.86	0.57	0.838	−2.50	0.77
Z Group 3	Z Group 0	4.58 *	0.48	0.000	3.21	5.96
	Z Group 1	0.56	0.60	0.838	−1.17	2.28
	Z Group 2	0.86	0.57	0.521	−0.77	2.50

* *p* < 0.05, Z: impedance, group 0 = VAS 0–2, group 1 = VAS 4–5, group 2 = VAS 6–7, group 3 = VAS 8.

**Table 10:** Analysis of phase angle value differences by VAS group.

VAS Group (I)	VAS Group (J)	Mean Difference (I-J)	SD	*p*-Value	95% Confidence Interval	
					Lower Bound	Upper Bound
PA Group 0	PA Group 1	−0.06 *	0.01	0.00	−0.08	−0.03
	PA Group 2	−0.06 *	0.01	0.00	−0.08	−0.04
	PA Group 3	−0.07 *	0.01	0.00	−0.09	−0.05
PA Group 1	PA Group 0	0.06 *	0.01	0.00	0.03	0.08
	PA Group 2	0.00	0.01	0.99	−0.03	0.02
	PA Group 3	−0.01	0.01	0.71	−0.04	0.02
PA Group 2	PA Group 0	0.06 *	0.01	0.00	0.04	0.08
	PA Group 1	−0.00	0.01	0.99	−0.02	0.03
	PA Group 3	−0.01	0.01	0.86	−0.03	0.02
PA Group 3	PA Group 0	0.07 *	0.01	0.00	0.05	0.09
	PA Group 1	0.10	0.01	0.71	−0.02	0.04
	PA Group 2	0.08	0.01	0.86	−0.02	0.03

* *p* < 0.05, PA: phase angle. Group 0 = VAS 0–2, group 1 = VAS 4–5, group 2 = VAS 6–7, group 3 = VAS 8.

**Table 11:** Comparison of ROC curve accuracy for BIP variables.

	Current	Frequency	Subject (N)		ROC Curve Accuracy		
			Healthy	LBP	R	Z	PA
Our Study	50 mA	182 Hz	45	40	0.981	0.982	0.962
[17] Wang et al. (2022)	1 mA	50 kHz	21	51	N/A	0.5898	0.6095
[18] Wang et al. (2019)	N/A	100 kHz	86	47	0.931	N/A	0.548

R: resistance, Z: impedance, PA: phase angle.

**Table 12:** Comparison of BIP variables.

	Healthy BIP			LBP BIP		
	R	Z	PA	R	Z	PA
Our Study	8.97 Ω	29.92 Ω	0.09	11.55 Ω	33.97 Ω	0.15
[17] Wang et al. (2022)	N/A	3.48 KΩ	1.06	N/A	6.09 KΩ	1.01
[18] Wang et al. (2019)	N/A	N/A	11.4	N/A	N/A	9.3

## Data Availability

The data presented in this study are available on request from the corresponding author due to privacy restrictions.
